# Acute weight gain, gender, and therapeutic response to antipsychotics in the treatment of patients with schizophrenia

**DOI:** 10.1186/1471-244X-5-3

**Published:** 2005-01-13

**Authors:** Haya Ascher-Svanum, Michael Stensland, Zhongyun Zhao, Bruce J Kinon

**Affiliations:** 1 Lilly Research Laboratories, Eli Lilly and Company, Indianapolis, Indiana

## Abstract

**Background:**

Previous research indicated that women are more vulnerable than men to adverse psychological consequences of weight gain. Other research has suggested that weight gain experienced during antipsychotic therapy may also psychologically impact women more negatively. This study assessed the impact of acute treatment-emergent weight gain on clinical and functional outcomes of patients with schizophrenia by patient gender and antipsychotic treatment (olanzapine or haloperidol).

**Methods:**

Data were drawn from the acute phase (first 6-weeks) of a double-blind randomized clinical trial of olanzapine versus haloperidol in the treatment of 1296 men and 700 women with schizophrenia-spectrum disorders. The associations between weight change and change in core schizophrenia symptoms, depressive symptoms, and functional status were examined post-hoc for men and women and for each medication group. Core schizophrenia symptoms (positive and negative) were measured with the Brief Psychiatric Rating Scale (BPRS), depressive symptoms with the BPRS Anxiety/Depression Scale and the Montgomery-Asberg Depression Rating Scale, and functional status with the mental and physical component scores on the Medical Outcome Survey-Short Form 36. Statistical analysis included methods that controlled for treatment duration.

**Results:**

Weight gain during 6-week treatment with olanzapine and haloperidol was significantly associated with improvements in core schizophrenia symptoms, depressive symptoms, mental functioning, and physical functioning for men and women alike. The conditional probability of clinical response (20% reduction in core schizophrenia symptom), given a clinically significant weight gain (at least 7% of baseline weight), showed that about half of the patients who lost weight responded to treatment, whereas three-quarters of the patients who had a clinically significant weight gain responded to treatment. The positive associations between therapeutic response and weight gain were similar for the olanzapine and haloperidol treatment groups. Improved outcomes were, however, more pronounced for the olanzapine-treated patients, and more olanzapine-treated patients gained weight.

**Conclusions:**

The findings of significant relationships between treatment-emergent weight gain and improvements in clinical and functional status at 6-weeks suggest that patients who have greater treatment-emergent weight gain are more likely to benefit from treatment with olanzapine or haloperidol regardless of gender.

## Background

Because antipsychotic drugs are considered the core treatment modality for schizophrenia [1] the differences among antipsychotics in terms of effectiveness, safety, and tolerability have expectedly become a topic of growing clinical and research interest [2]. The differences among antipsychotics in adverse events have garnered particular interest, with treatment-emergent weight gain becoming a focal point of attention and concern because weight gain can be associated with medical conditions such as type II diabetes, hypertension, and coronary artery disease [3]. Previous research has shown that there are variations with respect to the magnitude and the course of typical weight gain experienced during treatment with different antipsychotics [4]. Generally, the first generation antipsychotics, such as haloperidol, are associated with less weight gain than the second-generation antipsychotics. The newer atypical agents vary such that clozapine and olanzapine are associated with the greatest potential for weight gain, followed by risperidone, quetiapine, ziprasidone, [5] and aripiprazole.

Although most of the literature on treatment-emergent weight gain tends to focus on this event as adverse, a growing body of research has demonstrated a significant link between beneficial therapeutic response and treatment-emergent weight gain. With the exception of a few studies that failed to find an association between weight gain and better clinical outcome [6-9], most studies, primarily on clozapine, suggest an association between weight gain and better clinical outcome [10-19]. This expanding body of evidence augments studies on first-generation antipsychotics predating the introduction of atypical antipsychotics by about 30 years, also suggesting a link between weight gain and improved therapeutic response [20-22]. One study [17] reported mixed findings, where the association between treatment-emergent weight gain and clinical outcome was found for patients treated with clozapine and olanzapine, but not with risperidone or haloperidol, suggesting that this phenomenon may be specific to particular antipsychotics.

The study of gender differences in the relationship between treatment-emergent weight gain and therapeutic response has gained limited attention and provided conflicting results. A brief report on weight gain during clozapine therapy indicated that greater weight gain was associated with clinical improvement among women, but not among men [13]. In contrast, a more extensive analysis [16] demonstrated that clozapine-emergent weight gain predicted improvement in psychopathology among both men and women. It is unclear if there are gender differences in the association between treatment-emergent weight gain and therapeutic response, and if such gender differences exist, whether they are limited to a specific antipsychotic such as clozapine.

Women in the general population appear to be vulnerable to the adverse emotional and psychosocial consequences of weight gain. For women, obesity has been linked to lower life satisfaction, increased social isolation [23], and lower levels of psychological and physical functioning [24-26]. Compared to men, women are more likely to perceive themselves as overweight [27], to diet [28], and to participate in weight loss programs [28]. Based on generalizations from studies on women in the general population, several authors have speculated that antipsychotic-emergent weight gain will be similarly accompanied by negative psychological consequences [29-31], which will negatively impact women's response to antipsychotic therapy. However, it is unclear if women who gain weight during treatment with antipsychotics tend to experience adverse emotional consequences similar to those noted among women who gain weight in the general population. It is also unclear whether the association between treatment-emergent weight gain and clinical response differs by patient gender and by type of antipsychotic.

The primary objective of this study was to expand on prior research and investigate whether the relations between acute weight gain during antipsychotic therapy and treatment outcomes differ based on patient gender and the specific antipsychotic used in the treatment regimen, olanzapine or haloperidol. This study also aimed to broaden the definition of therapeutic response by extending beyond positive and negative symptoms of schizophrenia to depressive symptoms and levels of mental and physical functioning, because these domains tend to deteriorate with weight gain among women in the general population.

## Methods

### Subjects and study design

We used data of 1296 men and 700 women who participated in a randomized, double-blind, multi-center, clinical trial comparing olanzapine to haloperidol [32]. Participants met DSM-III-R criteria for schizophrenia spectrum disorders (schizophrenia, schizoaffective disorder, or schizophreniform disorders), and were required to have a total score on the Brief Psychiatric Rating Scale (BPRS) [33] of ≥ 18 and/or intolerance to current antipsychotic therapy, excluding haloperidol. Following approval of institutional review boards, written informed consent was obtained from all participants.

Participants were randomly assigned in 2:1 ratio (2 olanzapine subjects for each haloperidol subject). Although randomization was not stratified on gender or any other patient characteristics, it resulted in a 2:1 ratio for males (870/426) and for females (426/233). The olanzapine group (N = 1337) included 467 women and 870 men, and the haloperidol group (N = 659) was comprised of 233 women and 426 men.

Participants were randomly assigned to olanzapine, 5 to 20 mg/day, or haloperidol, 5 to 20 mg/day. The type of antipsychotic medication used prior to enrollment was not assessed in the current study, but the likelihood of previous treatment with an atypical antipsychotic drug was very low, because the study was initiated in 1994 when only clozapine was available in some of the sites. Further, randomization worked for patient and illness characteristics [32], and there is no reason to expect that the randomization did not work for other characteristics such as type of prior antipsychotic medication.

We used data from the acute phase, the first 6 weeks of the study, for several reasons. First and foremost, this study was a 6-week randomized double blind clinical trial with a 46-week "responder maintenance period", in which only patients who responded to the acute 6-week treatment per predetermined response criteria were eligible to continue. Consequently, the study design did not permit a longer-term analysis on the link between weight gain and improvement because only patients who improved during the first 6-weeks phase were followed-up for a longer time period. Second, the 6-week period represents a relevant time frame often used in clinical practice to determine treatment outcome and decide on treatment discontinuation [15]. For many clinicians the initial 6-weeks of antipsychotic therapy is a minimal time period in which to critically evaluate how patients are responding to a new course of therapy. Third, the rate of weight gain previously reported on clozapine was greatest in the first 6 weeks and slowed thereafter [16], such that the increase between 6 weeks and 6 months was equivalent in magnitude between baseline and 6 weeks. This observation further enhanced the relevance of studying this phenomenon during the first 6-weeks of treatment. And lastly, the short duration of the current study is comparable to most previous studies of antipsychotic-emergent weight gain and clinical improvement, thus enabling more direct comparisons between the present and the previous findings.

During the 6-week acute phase, the mean modal dose was 13.2 mg/day (SD = 5.8) for olanzapine and 11.8 mg/day (SD = 5.6) for haloperidol. There were no discontinuations due to weight gain as an adverse event for any treatment group during the 6-week study period, and the rate of discontinuation for any cause was similar for women (62.7%) and men (60.8%), with a significantly smaller proportion of the patients in the olanzapine group (33.5%) than in the haloperidol group (53.2%, p < .001). In addition, the percentage of patients who discontinued treatment because of an adverse event or a lack of efficacy was significantly higher in the haloperidol group than in the olanzapine group. Further details on the parent study design and primary findings are available elsewhere [32].

### Measures

This investigation used measures of positive and negative symptoms ("core schizophrenia symptoms"), depressive symptoms, functional status, and body weight. Core symptoms of schizophrenia were assessed by the Positive Symptom and the Negative Symptom subscales on the BPRS (scored on a scale of 0–6) extracted from the Positive and Negative Syndrome Scale (PANSS) [34]. Levels of depressive symptoms were assessed by the Depression/Anxiety subscale in the BPRS and total score on the Montgomery-Asberg Depression Rating Scale (MADRS) [35]. The Physical and the Mental Component scores on The Medical Outcome Survey – Short Form 36 (SF-36) [36] assessed physical and mental functioning. The SF-36 provides scores on eight functional scales: physical functioning, role limitations due to physical functioning, bodily pain, general health, social functioning, role limitations due to emotional problems, vitality and mental health. The first four scales can be summarized into a Physical Component Score (PCS) and the latter four constitute the Mental Component Score (MCS). PCS and MCS are often used alone because they account for 85% of reliable variance of the eight SF-36 domains, without losing information. It is notable that unlike the symptom measures, which were clinician-rated scales, the SF-36 is a patient-reported measure that provides patients' subjective appraisal of current functional status independently of clinicians' perceptions. Weight change (in kilograms) was measured from baseline to 6 weeks, or to endpoint for patients who dropped out of the study prior to the 6-week visit. BMI was calculated as weight in kilograms divided by the square of height in meters (BMI = kg/m^2^).

To enhance comparability of findings on different measures, the clinical measures were all standardized to z-scores. For the BPRS Core Symptoms, MADRS, and BPRS Anxiety and Depressive Subscale, this was done by subtracting the measure's overall mean and dividing by the measure's standard deviation at baseline. A single measure of depressive symptoms was calculated as the average of the standardized MADRS and BPRS Anxiety and Depressive Subscale. If a score was missing on either depression measure, the score on the available measure was used. The two depression measures were pooled because each is an independent and valid estimate of patients' level of depressive symptoms, and aggregating them should provide the best and most comprehensive estimate of depressive symptoms. Additionally, the pooling helped minimize loss of data, which are assumed not to be missing at random. The SF-36 Physical and Mental Component scores were converted from T-Scores to z-scores.

### Statistical analysis

Baseline comparisons used independent samples t-tests for continuous variables and chi-square tests for categorical variables. Effects of treatment and gender on independent variables were assessed using ANCOVA, with the baseline score as well as the number of weeks in the study as covariates. The relationship between change in weight and change in each outcome variable was assessed using separate multiple linear regression analyses, each with corresponding clinical change score as a dependent variable, the corresponding baseline score and number of weeks in the study as covariates, and the following independent variables: weight change, treatment group assignment, and gender. In an additional analysis, the interactions of these three independent variables were added to the regression models.

The analyses included measures from baseline and the 6-week visit. Missing data were handled by carrying forward the last observation for all patients with at least one post-baseline assessment. All analyses were performed using the Statistical Package for the Social Sciences (SPSS) version 11.0.

## Results

### Baseline characteristics

Relative to men, women were older, more likely to be Caucasians, were more likely to be overweight or obese, had less severe positive symptoms, lower levels of physical functioning, and had higher levels of depressive symptoms (Table 1). Women were also more likely to be diagnosed with a schizoaffective disorder and less likely to be diagnosed with schizophrenia. At baseline, the weight and BMI of the haloperidol-treated women and men, were significantly greater than the weight and BMI of olanzapine-treated women and men (Table 2). Further, men in either medication group weighed significantly more than women at baseline, on the average, and their mean baseline BMI was significantly lower than that of women.

**Table 1:** Gender differences at baseline*

	All patients	Women	Men	
Characteristic	N = 1996	N = 700	N = 1296	P
Demographics				
Age	38.6 (11.4)	40.9 (12.8)	37.3 (10.4)	<0.001
Race				0.013
Caucasian	1600 (80.2%)	587 (83.9%)	1013 (78.2%)	0.002
African descent	220 (11.0%)	67 (9.6%)	153 (11.8%)	0.128
Hispanic	83 (4.2%)	19 (2.7%)	64 (4.9%)	0.018
Other	93 (4.7%)	27 (3.9%)	66 (5.1%)	0.211
Diagnosis				<0.001
Schizophrenia	1658 (83.1%)	526 (75.1%)	1132 (87.3%)	<0.001
Schizoaffective disorder	300 (15.0%)	157 (22.4%)	143 (11.0%)	<0.001
Schizophreniform disorder	38 (1.9%)	17 (2.4%)	21 (1.6%)	0.207

Core schizophrenia symptoms				
BPRS total	33.4 (10.7)	33.5 (11.2)	33.3 (10.5)	0.660
BPRS positive	10.3 (4.1)	10.0 (4.1)	10.5 (4.0)	0.017
BPRS negative	6.7 (3.3)	6.7 (3.4)	6.7 (3.3)	0.791

Depressive symptoms				
MADRS	16.6 (8.8)	17.3 (9.3)	16.3 (8.5)	0.031
BPRS anxiety and depression	7.5 (3.8)	8.0 (3.9)	7.3 (3.8)	<0.001

Functional status				
SF-36 physical component	43.6 (13.0)	41.4 (14.0)	44.5 (12.5)	0.004
SF-36 mental component	34.6 (12.4)	34.6 (12.9)	34.6 (12.2)	0.958

Weight				
Weight, kg	76.8 (17.1)	70.2 (16.4)	80.4 (16.3)	<0.001
BMI	26.0 (5.2)	26.5 (5.8)	25.8 (4.9)	0.007
BMI level				<0.001
Underweight to average (BMI < 25)	930 (48.9%)	319 (47.6%)	611 (49.6%)	0.418
Overweight (BMI ≥ 25 and < 30)	609 (32.0%)	191 (28.5%)	418 (33.9%)	0.016
Obese (BMI ≥ 30)	364 (19.1%)	160 (23.9%)	204 (16.5%)	<0.001

* Data are presented as Mean (SD) or N (%).

P-values refer to differences between women and men.

**Table 2:** Outcomes by gender and treatment group

	Women				Men			
	
	Olanzapine		Haloperidol		Olanzapine		Haloperidol	
Outcome measure	Baseline	Endpoint	Baseline	Endpoint	Baseline	Endpoint	Baseline	Endpoint

Core symptoms								
BPRS positive	10.1 (4.0)	6.5 (4.8)	9.9 (4.3)	6.9 (4.4)	10.3 (4.1)	7.0 (4.6)	10.7 (4.0)	8.0 (4.6)
BPRS negative*	6.6 (3.3)	4.4 (3.1)	6.8 (3.4)	5.5 (3.3)	6.7 (3.2)	4.8 (2.9)	6.8 (3.5)	5.5 (3.2)
Depressive symptoms								
MADRS*	17.8 (9.6)	11.0 (9.3)	16.1 (8.6)	13.5 (10.7)	16.0 (8.4)	10.5 (7.7)	17.0 (8.7)	13.7 (9.4)
BPRS anxiety & depression*	8.1 (3.8)	5.0 (4.0)	7.8 (4.1)	6.0 (4.3)	7.1 (3.7)	4.5 (3.6)	7.7 (3.9)	5.7 (3.9)
Functioning								
SF-36 mental component*	34.7 (13.1)	41.8 (12.1)	34.2 (12.4)	36.5 (13.0)	34.6 (12.4)	40.6 (12.0)	34.7 (11.7)	37.8 (12.3)
SF-36 physical component*	41.3 (14.3)	45.0 (13.9)	41.9 (13.5)	42.1 (13.8)	44.7 (12.2)	48.7 (11.6)	43.9 (13.2)	45.4 (13.1)
Weight								
Weight, kg* ^†^	68.9 (15.5)	70.5 (15.8)	72.6 (17.8)	72.4 (17.6)	80.3 (15.9)	82.5 (16.2)	80.7 (17.1)	81.0 (17.4)
BMI* ^†^	26.1 (5.4)	26.7 (5.5)	27.2 (6.6)	27.1 (6.5)	25.8 (4.8)	26.5 (4.9)	25.7 (5.0)	25.8 (5.0)

* Therapy effect (p < .05), reflecting significant differences between olanzapine and haloperidol-treated patients at baseline.

^† ^Gender effect (p < .05), reflecting significant differences between women and men on weight parameters within each treatment group at baseline, and between women and men when combined baseline values across treatment groups.

### Weight gain by treatment group and gender

**Figure 1:**
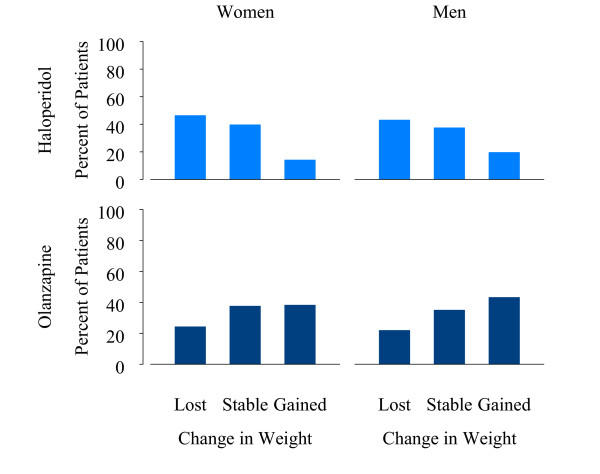
Percentage of patients with different levels of weight change by gender and medication. Patients were placed in 3 equal groups based on their percent change in weight: "Lost" indicates any weight loss, "Stable" indicates ≥ 0% to <3% weight gain, and "Gained" indicates ≥ 3% weight gain. Olanzapine treatment group (N = 1337; 870 men, 467 women); Haloperidol treatment group (N = 659, 426 men, 233 women).

In order to illustrate the differences in weight gain by treatment group and gender, the patients were grouped into thirds based on their percentage of change in weight from baseline. Approximately one third of all patients (29.8%) lost weight (any decrease), one third (36.6%) had relatively stable weight (0% to <3% increase), and one-third (33.6%) gained weight (≥ 3% increase). The corresponding mean weight changes in kilograms were -2.1 kg, 0.9 kg, and 4.6 kg, for lost, stable, and increased weight groups, respectively. Figure 1 demonstrates that men and women had a similar weight gain pattern within each treatment group, and that 59% of olanzapine-treated patients and 82% of haloperidol-treated patients either lost weight or maintained stable weight. Further, 17.6% of the haloperidol treatment group and 41.4% of the olanzapine-treated patients gained at least 3% of their baseline body weight. Compared to the haloperidol-treated patients, the olanzapine treatment group had a greater increase in absolute weight (0.3 kg vs. 2.0 kg, F(1,1901) = 122.0, p < 0.001) and a significantly greater proportion of patients with a potentially clinically meaningful weight gain, defined as an increase of at least 7% from baseline body weight (3.0% vs. 13.6%, χ^2^(1, N = 1913) = 51.8, p < 0.001).

Compared to women, men experienced greater increases in absolute weight (0.9 kg vs.1.5 kg F(1,1901) = 17.3, p < 0.001), were more likely to experience greater increases in BMI (0.35 vs. 0.48; F(1,1889) = 5.8, p = 0.016), and were more likely to have an increase of at least 7% from baseline body weight (8.1% vs.11.2%; χ^2^(1, N = 1913, p = 0.032). Within the olanzapine treatment group, but not the haloperidol treatment group, significantly more men than women experienced a potentially clinically meaningful weight gain (11.0% vs.15.0%; χ^2^(1, N = 1286) = 4.0, p = 0.045 for women and men in the olanzapine treatment group, and 2.3% vs. 3.5%; χ^2^(1, N = 627) = 0.7, p = 0.40, for women and men in the haloperidol treatment group).

### Outcomes by treatment group and gender

Table 2 presents the outcome measures and BMI by gender and by treatment group at baseline and endpoints. As previously documented in the parent study [32], there were treatment effects on these outcome measures such that olanzapine-treated patients showed greater improvements than the haloperidol treatment group. There were no gender effects for any of the clinical (core symptoms of schizophrenia, depressive symptoms) and functional outcome measures (mental and physical functioning).

### Outcomes, weight change, and gender

To assess the potential effects of gender on the relationship between outcomes and weight change we performed a set of regression analyses predicting change in each of the outcome variables (i.e., Core symptoms of schizophrenia, depressive symptoms, gender, and the interaction of these variables). The results indicated that gender was not a significant variable (i.e., the following components were not significant in any of the analyises: gender, gender by weight change, gender by treatment group, and gender by treatment group by weight change). Therefore, gender was dropped from subsequent analyses.

Since men and women were not found to significantly differ on any of the clinical outcome measures and had a similar pattern of weight gain within each treatment group, we examined the association between weight change and change in treatment outcomes for all patients within each treatment group. Regression analyses demonstrated that for both olanzapine and haloperidol-treated patients, increases in weight were significantly associated with improvements in core schizophrenia symptoms, (*B *= -0.038, t(1899) = 5.6, p < 0.001), in depressive symptoms (*B *= -0.030, t(1899) = 5.3, p < 0.001), in mental functioning (*B *= 0.026, t(700) = 2.0, p = 0.047), and in physical functioning (*B *= 0.028, t(700) = 2.3, p = 0.021). Because level of depressive symptoms was based on two depression measures, the MADRS and the BPRS depression/anxiety subscale, we repeated the analysis using each of these measure separately. Results were unchanged.

The regression coefficients (*B*'s) indicated that every one- kilogram increase in weight at 6-weeks was associated with approximately 0.03 standard deviations improvement in each clinical outcome parameter, when controlling for the effects of treatment group, gender, treatment group-gender interaction, baseline weight, the corresponding baseline outcome measure, and the number of weeks in the study.

**Figure 2:**
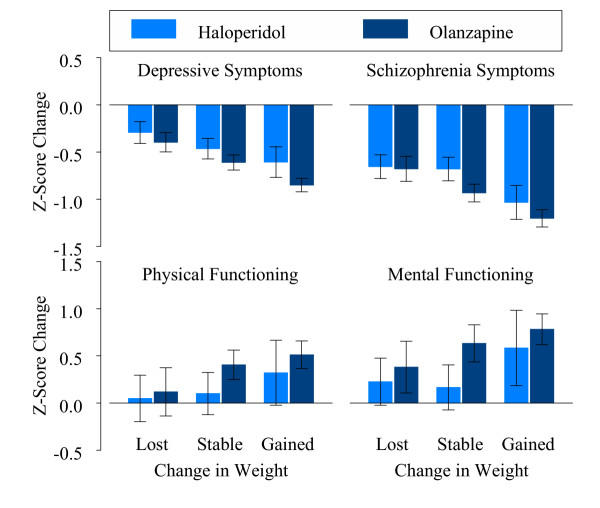
Change in outcomes by change in weight and treatment group for all patients. Patients were grouped in thirds based on their percent change in weight: "Lost" indicates any weight loss, "Stable" indicates 0% to <3% weight gain, and "Gained" indicates weight gain of 3% or more. "Depression" as measured by the MADRS or BPRS anxiety and depression scale. "Schizophrenia Symptoms" as measured by the BPRS positive symptoms and BPRS negative symptoms scales. "Physical Functioning" as measured by the SF-36 physical component score. "Mental Functioning" as measured by the SF-36 mental component score. Error bars represent 95% confidence intervals.

In order to graphically illustrate the findings, the patients were grouped into thirds based on their percent change in weight as described above, resulting in lost, stable, and increased weight groups. Figure 2 demonstrates the similarity in the relationships between weight changes and changes in the four treatment outcome variables for the olanzapine and haloperidol treatment groups.

Consistent with a prior analytical approach by Czobor and colleagues [17], we also performed analyses (ANCOVA, controlling for baseline weight and weeks in the study) that specifically contrasted patients who demonstrated clinical improvement (reduction in BPRS Core Symptoms > 20%) with those who deteriorated by any amount. On core symptoms, improved olanzapine patients gained 2.49 kg, compared with 1.42 kg for those who deteriorated (F(1,1278) = 14.7, p < 0.001). Improved patients on haloperidol gained 0.08 kg while those who deteriorated lost 0.44 kg (F(1, 617) = 2.9, p = 0.087). In order to directly compare current findings with those previously reported by Czobor and associates, we repeated the analysis using their analytical variables (absolute weight change (kg) and PANSS total score), while covarying baseline bodyweight and baseline PANSS total score. This analysis demonstrated that improved patients on olanzapine gained 2.37 kg, compared with 0.59 kg for those who deteriorated (F(1,1278) = 53.1, p < 0.001). Improved patients on haloperidol gained 0.15 kg, while deteriorated patients on haloperidol lost 0.55 kg (F(1,618) = 6.7, p = 0.010). Results were similar when also controlling for weeks in the study. The partial correlations between weight gain (kg) and therapeutic response as measured by the PANSS total score (controlling for baseline weight, baseline PANSS total score, and weeks of treatment) were statistically significant for the olanzapine and haloperidol treatment groups (partial r = -0.15, N = 1279, p < 0.001 for olanzapine; partial r = -0.11, N = 618, p = 0.006 for haloperidol). When using the Czobor and associates method (controlling only for baseline weight and baseline PANSS total score), the partial correlations were more disparate across treatment groups (partial r = -0.24, N = 1280, p < 0.001 for olanzapine; partial r = -0.10, N = 619, p = 0.013 for haloperidol), highlighting the importance of controlling for weeks of treatment. These correlations were similar in direction but of smaller magnitude than the partial correlations reported by Czobor and associates (partial r = -0.57, df = 37, p < 0.001 for olanzapine; partial r = - 0.30, df = 35, p = 0.060 for haloperidol).

Although weight gain was identified as a prognostic marker of therapeutic response for both treatment groups, it was unclear if this marker is stronger for the olanzapine than the haloperidol treatment group because the olanzapine-treated patients had greater weight gain and greater therapeutic improvements compared to the haloperidol treatment group. To address this question, we calculated the conditional probability of clinical response, defined as reduction in BPRS core symptoms > 20%, given that the patient experienced various amounts of weight gain on olanzapine and on haloperidol. Results in Table 3 demonstrate that weight gain was a similar prognostic indicator for each treatment group, as patients who gained more weight were significantly more likely to respond to treatment for both treatment groups. About half of the patients who lost weight responded to treatment, whereas three-quarters of patients who had a clinically significant weight gain (≥7%) responded to treatment.

**Table 3:** Conditional probability of response given different amounts of weight gain for all patients and by medication

	Olanzapine^a^					Haloperidol^b^				
	N = 1283					N = 622				
	
Weight change	Did not respond		Responded^c^		P(R| W)	Did not respond		Responded^c^		P(R| W)
	N	%	N	%		N	%	N	%	
Lost weight (< 0%)	210	16.4%	187	14.6%	.47	176	28.3%	166	26.7%	.49
Gained 0 to < 3%	118	9.2%	234	18.3%	.67	88	14.2%	83	13.3%	.49
Gained 3 to < 7%	111	8.7%	248	19.3%	.69	39	6.3%	51	8.2%	.57
Gained ≥ 7%	43	3.4%	132	10.3%	.75	5	0.8%	14	2.3%	.74

*Note. *Response was defined as a greater than 20% decrease in BPRS Core Symptoms

^a ^Mantel-Haenszel test of linear by linear association, *χ *^2^(1, N = 1283) = 52.3, p < 0.001.

^b ^Mantel-Haenszel test of linear by linear association, *χ *^2^(1, N = 622) = 4.1, p = 0.044.

^c^Probability of response given level of weight change.

## Discussion

Like women in the general population, women with schizophrenia were more likely to be obese [25], depressed [37], and to physically function at a poorer level than men [26]. Despite these similarities, which would be expected to bode poorly for the effects of acute weight gain on women's treatment outcomes, women and men who gained weight during antipsychotic therapy demonstrated significant improvements on core schizophrenia symptoms, depressive symptom, and mental and physical level of functioning. Overall, weight gain was found to be linked to better clinical response among men and women treated with olanzapine or haloperidol. This link impacted olanzapine-treated patients more than those treated with haloperidol because improved clinical and functional outcomes were more pronounced for the olanzapine-treated patients, who were also more likely to experience weight gain than patients treated with haloperidol. The current study adds to the literature by demonstrating a positive association between treatment-emergent weight gain and better clinical outcomes that extends beyond positive and negative symptoms to depressive symptoms and functional status. Depressive symptoms in schizophrenia are known to be a distinctive clinical dimension of prognostic significance [38] that is associated with compromised quality of life [39], increased risk of psychotic relapse [40], suicidal tendencies, work impairment, lower activity, worse daily functioning, and poorer life satisfaction [41]. In this study, weight gain during antipsychotic therapy was linked to improvements in both core symptoms of schizophrenia and in depressive symptoms, two distinct and clinically meaningful dimensions of outcome in the treatment of schizophrenia.

Current findings are consistent with previous research [10-19] and provide further support to the hypothesis [16] that a positive link between treatment-emergent weight gain and improved clinical response may be a generalized phenomenon across antipsychotic medications. Although a recent study [17] demonstrated this phenomenon for clozapine and olanzapine-treated patients but not in the haloperidol or risperidone treatment groups, examination of its findings revealed great similarity to the current results, with a moderate association between weight gain and therapeutic response for the haloperidol treatment group despite a small sample size. Interestingly, the size of the effects reported by Czobor and associates (partial correlations of -0.57 for olanzapine and -0.30 for haloperidol) were numerically larger than those found in the current study (partial correlations of -0.24 for olanzapine and -0.10 for haloperidol). Our study supports the findings of Czobor and associates but with sufficient statistical power to produce statistically significant results for both the olanzapine and the haloperidol treatment groups.

Although the current results are consistent with those reported in a number of previous prospective studies, our findings are incongruent with two retrospective surveys. In the more recent study [42], self-administered surveys were distributed to schizophrenia patients through chapters of the National Alliance for the Mentally Ill to assess their perceptions about the negative impact of treatment-emergent weight gain on psychosocial functioning. The authors concluded that weight gain is directly associated with reduced quality of life. Several limitations of this study were previously noted [43], pointing particularly to a major confounding factor: most of the respondents started their antipsychotic medications several years before the survey. Because antipsychotics differ in the magnitude and in the trajectory of weight gain over time [44], the reported differences may reflect differences between a group of patients whose illness is well managed and thus are reporting a sense of relative psychological well being and a group of distressed patients who are adjusting to a new antipsychotic regimen [43]. The other survey [45] queried depressed psychotic patients who called a mental health crisis line about the impact of eight adverse events, including weight gain, on their emotional distress and satisfaction with treatment. Although weight gain was the adverse event reported least, it was viewed as the most distressing, particularly for women, and was linked to lower satisfaction with treatment. This survey, which was noted for its lack of rigorous design [2], did not report the treatment duration on the antipsychotic drugs. Resultantly, the respondents may have started the antipsychotic regimens years before the survey, obscuring the findings in a manner similar to that in the survey by Allison and colleagues [42].

In essence, the two retrospective self-reports appear to have assessed patients' treatment satisfaction and perceptions rather than objective parameters of clinical change and treatment progress. Studies that objectively measure weight change and clinical response in a prospective fashion are more desirable as they provide more objective information. This is especially important because retrospective self-reports may capture the social climate rather than objective changes in clinical outcomes. It is noteworthy, however, that there are four prospective studies reporting findings that are inconsistent with ours [6-9]. The reasons for the inconsistencies are not clear but may be due to small sample size. The sample size needed to detect a correlation of .20 with 80% power is 194, while sample sizes for these four studies ranged from 30 to 82.

Although the current study found a link between treatment-emergent weight gain and better therapeutic response, its correlational nature does not allow for discerning the underlying causes. There are numerous factors and poorly understood mechanisms that may impact patients' weight gain during treatment, including environmental, behavioral, neurochemical, genetic, and clinical factors [17]. It was previously noted, for example, that the association between weight gain and therapeutic improvement may reflect for some patients the restoration of body weight lost during an acute episode because patients were previously found to restore their original body weight upon recovery, even prior to the introduction of antipsychotics [46].

While the association between treatment-emergent weight gain and therapeutic response may be due to specific pharmacological pathways, it is also possible that non-pharmacological pathways play an important role. The link between weight gain and therapeutic response may be an epiphenomenon that accompanies clinical and functional improvements by influencing patients' increased motivation, pleasures, and specific behaviors that enhance weight gain [16]. Future studies will be needed to evaluate the correlations reported here in order to better understand the underlying mechanisms of treatment-emergent weight gain, and help differentiate pharmacological from non-pharmacological pathways to weight gain in the treatment of schizophrenia with antipsychotics. A promising research strategy [47] may involve the use of placebo-controlled trials of antipsychotics to contrast weight change between patients who improved on placebo with those who deteriorated on placebo. It is important to note that regardless of the pathways to weight gain, the link between excess weight and greater morbidity and mortality calls for careful clinical attention during the treatment of patients with schizophrenia.

Another important issue that needs addressing is the growing focus on the associations between obesity and poorer quality of life among schizophrenia patients [48]. Such research helps highlight the need to distinguish between treatment-emergent weight gain and obesity. Although these terms are not mutually exclusive, they are not synonymous either. Gaining weight during treatment with antipsychotics should not be equated with becoming obese. For example, thin individuals may gain a potentially clinically meaningful proportion of their baseline body weight (≥ 7%) and attain an average BMI, whereas obese individuals may gain the same proportion of their baseline weight but maintain their initial obese status per BMI categorization. There are numerous permutations to this phenomenon, suggesting the need to recognize its complexity and pursue further studies that may help clarify the causes and the consequences of treatment-emergent weight gain among individuals with schizophrenia who differ in their baseline body weight.

The current study has its limitations. First, it examined weight change post-hoc and only during the acute phase of the illness, which was confined to the first 6 weeks of treatment, and the findings may not generalize to long-term treatment-emergent weight gain. It is noteworthy that this study assessed treatment-emergent weight gain at 6-weeks, although patients continue to accrue weight beyond the acute treatment phase. For olanzapine-treated patients, the mean weight gain observed at 6-weeks (2.0 kg) was about a third of 6.26 kg mean weight gain found at 39 weeks, when weight gain tends to plateau on olanzapine [49]. Similarly, the haloperidol-treated patients had a 0.3 kg mean weight gain at 6 weeks, which was less than half of 0.69 kg mean weight gain observed for these patients after 39 weeks of treatment. This observation highlights the need to assess the association between weight gain and treatment outcomes in longer-term studies. The choice of 6-weeks was not only driven by the design of the study, in which treatment responders in the acute phase were followed up in the 46-week maintenance period of the study, but also by the clinical relevance of the acute treatment phase. Clinicians often use the first 6-weeks of treatment to assess the tolerability and effectiveness of a new antipsychotic regimen and to decide whether to continue or discontinue that course of therapy [15]. Further, the study of the first 6 weeks of treatment enabled comparisons of the current findings with other studies, which were typically of short-term duration.

Although weight gain appears to be greatest and most rapid during the first 6 weeks of treatment with clozapine [16], and during the first 12 weeks for olanzapine with a trend toward a plateau after approximately 39 weeks of treatment, [49] longer-term studies will be needed to determine the validity of the current findings in longer-term treatment. This may be, however, difficult to study. Patient attrition from studies is not random, with those experiencing poor treatment efficacy or poor tolerability being more likely to discontinue the study, leaving a relatively homogeneous group of study completers who are also treatment responders. Such reduction in the variability of treatment outcomes may diminish the likelihood of finding this phenomenon in long term randomized double blind studies. Further, if this phenomenon were to be investigated in long-term naturalistic observational studies, one would likely face another problem, namely the prevalent use of polypharmacy [50], and the dynamic nature of treatment for schizophrenia, [51] with frequent changes in antipsychotic regimens and in concomitant psychotropic medications. Such complexity may increase the difficult in identifying which treatment at what time was associated with which weight gain and treatment outcome.

It is of interest to note, however, that despite rapid weight gain during the 6-week period in our study, when weight gain is more likely to be noticed by the patients and their clinicians and thus may elicit a negative emotional response, the weight gain in this study was not only linked to improved clinical and functional status but also to reduced emotional distress as measured by the depression scales.

Another limitation of the study is its lack of assessment of patients' adherence with medication. It is possible that weight gain and improvement occurred together because improvements occur mostly in patients who are medication adherent. Although this was not assessed in the present study, this possibility was previously studied by Meltzer et al. [16], who found a significant association between clozapine-emergent weight gain and improved psychopathology. In their study, non-adherent patients were expected to have lower or absent plasma clozapine levels, but there was no relationship between plasma clozapine levels and weight gain or clinical response. Meltzer and colleagues also monitored adherence closely during weekly visits to determine white blood count and found no evidence of intermittent or poor adherence in their study patients. Additionally, the associations between weight gain and improved outcomes in our study were similarly found within the haloperidol and within the olanzapine treatment groups when controlling for treatment duration. Treatment duration is a proxy for time on the medication in randomized double-blind trials, where treatment discontinuation for any reason results in patient's discontinuation from the study. Thus, if patients were more adherent with one antipsychotic drug than with the other, and medication adherence influenced the associations between weight gain and outcomes, then one would expect to find the association between weight gain and improved outcomes to be present only in the more adherent treatment group, but not in both treatment groups, as found in the present study.

Another study limitation is the correlational nature of the analyses, which precludes cause-effect relationship and allows for the possibility that the observed associations might be due to an unobserved variable or set of variables. Further, the relatively low correlations suggest that the association explains only a small proportion of the variance in treatment outcomes. Response to antipsychotic medications is a complex phenomenon that is associated by numerous relatively independent components [16] and weight gain is only one of them. Nonetheless, this link was demonstrated when using other statistical approaches, including contrasting of weight gains between responders and patients who did not respond, by identifying the degree of improvement associated with every 1-kg gained at 6 weeks, and by calculating the conditional probability of therapeutic response given various amounts of weight gain. These findings are important as they suggest that acute weight gain is a valuable prognostic marker in the treatment of schizophrenia.

Next, because the study included patients with a moderately severe level of symptomatology, the current findings may not generalize to patients with milder or residual symptoms of schizophrenia. However, the relationships among the severity of patients' baseline symptomatology, treatment-emergent weight gain and therapeutic response is currently unclear. And lastly, this study used the SF-36, a self-report measure of functional status, which was not designed to assess the potential impact of weight gain on patients' functional status or quality of life. Preliminary information on the first measure designed to specifically capture the impact of antipsychotic-emergent weight gain on patients' psychosocial functioning was only recently published [52]. One would have expected, however, to detect a decline in patients' mental or physical levels of functioning if the experienced weight gain were to have adverse impact during the acute treatment phase.

## Conclusions

Women (and men) with schizophrenia who gained weight during treatment with olanzapine or haloperidol did not experience worsening of clinical or functional status. To the contrary, they had significant improvements in core symptoms of schizophrenia, depressive symptoms, and mental and physical level of functioning. Although excessive weight gain, regardless of origin, is of concern due to its association with physical health problems, the current findings suggest that patients who have greater treatment-emergent weight gain are more likely to benefit from treatment with olanzapine or haloperidol. Findings highlight the complexity inherent in medication management of schizophrenia patients and the need to balance treatment risks and benefits for each patient. In addition, further prospective studies will be required to assess the effects of weight gain, in both psychiatric and medical terms, on individuals treated for schizophrenia with various antipsychotic medications.

## Competing interests

The authors are employees of Eli Lilly and Company, Indianapolis, Indiana

## Authors' contributions

• HAS conceived of the study, participated in its design, the analytical plan, the interpretation of the results, and drafted the manuscript

• MS participated in the design of the study, the analytical plan, the interpretation of the results, and performed the statistical analysis

• ZZ and BK participated in the design of the study, the interpretation of the results, and the drafting of the manuscript.

## Pre-publication history

The pre-publication history for this paper can be accessed here:



## References

[B1] American Psychiatric Association (2004). Practice Guidelines for the Treatment of Patients with Schizophrenia, Second Edition. Am J Psychiatry.

[B2] Bagnall A-M, Jones L, Ginnelly L, Lewis R, Glanville J, Gillbody S, Davis L, Torgerson D, Kleijnen J (2003). A systematic review of atypical antipsychotic drugs in schizophrenia. Health Technol Assesss.

[B3] Lee IM, Paffenbarger RS (1992). Changes in body weight and longevity. JAMA.

[B4] Taylor DM, McAskill R (2000). Atypical antipsychotics and weight gain: a systematic review. Acta Psychiatr Scand.

[B5] Allison DB, Mentore JL, Heo M, Chandler LP, Cappelleri JC, Infante MC, Weiden PJ (1999). Antipsychotic-induced weight gain: A comprehensive research synthesis. Am J Psychiatry.

[B6] Umbricht DS, Pollack S, Kane JM (1994). Clozapine and weight gain. J Clin Psychiatry.

[B7] Hummer M, Kemmler G, Kurtz M, Kurtzthaler I, Oberbauer H, Fleischhacker WW (1995). Weight gain induced by clozapine. Eur Neuropsychopharmacol.

[B8] Bustillo JR, Buchanan RW, Isirh D, Breier A (1996). Differential effect of clozapine weight: a controlled study. Am J Psychiatry.

[B9] Poyurovsky M, Pashinian A, Gil-Ad I, Maayan R, Schneidman M, Fuchs C, Weizman A (2002). Olanzapine-induced weight gain in patients with first-episode schizophrenia: a double blind, placebo-controlled study of fluoxetine addition. Am J Psychiatry.

[B10] Leadbetter R, Shutty M, Pavalonis D, Vieweg V, Higgins P, Downs M (1992). Clozapine-induced weight gain: prevalence and clinical relevance. Am J Psychiatry.

[B11] Lamberti JS, Bellnier T, Schwarzkopf SB (1992). Weight gain among schizophrenic patients treated with clozapine. Am J Psychiatry.

[B12] Gupta S, Droney T, Al-Samarrai S, Keller P, Fran B (1999). Olanzapine: Weight gain and therapeutic efficacy. J Clin Psychopharmacol.

[B13] Bai YM, Lin CC, Chen JY, Lin CY (1999). Weight gain among patients on clozapine. Psychiatr Serv.

[B14] Ritter LM, Meador-Woodruff JH, Dalack GW (2000). Weight gain and response to olanzapine treatment in schizophrenia [abstract]. Biol Psychiatry.

[B15] Basson BR, Kinon BJ, Taylor CC, Szymanski KA, Gillmore JA, Tollefson GD (2001). Factors influencing acute weight change in patients with schizophrenia treated with olanzapine, haloperidol, or risperidone. J Clin Psychiatry.

[B16] Meltzer HY, Perry E, Jayathilake K (2002). Clozapine-induced weight gain predicts improvement in psychopathology. Schizophr Res.

[B17] Czobor P, Volavka J, Sheitman B, Lindenmaer JP, Citrome L, McEvoy J, Cooper TB, Chakos M, Lieberman JA (2002). Antipsychotic-induced weight gain and therapeutic response: A differential association. J Clin Psychopharm.

[B18] Garyfallos G, Dimelis D, Kounaiakis P, Sidiropoulos N, Karastergiou A, Lavrentiadis G, Giouzepas J, Fokas K (2003). Olanzapine versus risperidone: weight gain and elevation of serum triglyceride levels. European Psychiatry.

[B19] Lane HY, Chang YC, Cheng YC, Liu GC, Lin XR, Chang WH (2003). Effects of patient demographics, risperidone dosage, and clinical outcome on body weight in acutely exacerbated schizophrenia. J Clin Psychiatry.

[B20] Planansky K, Heilizer F (1959). Weight changes in relation to the characteristics of patients on chlorpromazine. J Clin Exp Psychopathol.

[B21] Klett CJ, Caffey EM (1960). Weight changes during treatment with phenothiazine derivatives. J Neuropsychiatr.

[B22] Singh MM, De Dios LV, Klein NS (1970). Weight as a correlate of clinical response to psychotropic drugs. Psychosomatics.

[B23] Sarlio-Lahteenkorva S (2001). Weight loss and quality of life among obese people. Social Indicators Research.

[B24] Rumpel C, Ingram DD, Harris TB, Madans J (1994). The association between weight change and psychological well being in women. Int J Obes Relat Metab Disord.

[B25] Le Pen C, Levy E, Loos F, Banzet MN, Basdevant A (1998). "Specific" scale compared with "generic" scale: a double measurement of the quality of life in a French community sample of obese subjects. J Epidemiol Community Health.

[B26] Stafford M, Hemingway H, Marmot M (1998). Current obesity, steady weight change and weight fluctuation as predictors of physical functioning in middle aged office workers: the Whitehall II Study. Int J Obes Relat Metab Disord.

[B27] Emslie C, Hunt K, Macintyre S (2001). Perceptions of body image among working men and women. J Epidemiol Community Health.

[B28] Neumark-Sztainer D, Sherwood NE, French SA, Jeffery RW (1999). Weight control behaviors among adult men and women: cause for concern?. Obes Res.

[B29] Kawachi I (1999). Physical and psychological consequences of weight gain. J Clin Psychiatry.

[B30] Blin O, Micallef J (2001). Antipsychotic-associated weight gain and clinical outcome parameters. J Clin Psychiatry.

[B31] Kurzthaler I, Fleischhacker WW (2001). The clinical implications of weight gain in schizophrenia. J Clin Psychiatry.

[B32] Tollefson GD, Beasley CM, Tran PV, Street JS, Krueger JA, Tamura RN, Graffeo KA, Thieme ME (1997). Olanzapine versus haloperidol in the treatment of schizophrenia and schizoaffective and schizophreniform disorders: results of an international collaborative trial. Am J Psychiatry.

[B33] Overall JE, Gorham DR (1962). The Brief Psychiatric Rating Scale. Psychol Rep.

[B34] Kay SR, Fiszbein A, Opler LA (1987). The Positive and Negative Syndrome Scale (PANSS) for schizophrenia. Schizophr Bull.

[B35] Montgomery SA, Asberg M (1979). A new depression scale designed to be sensitive to change. Br J Psychiatry.

[B36] Ware JE, Sherbourne CD (1992). The MOS 36-item short-form health survey (SF-36). I. Conceptual framework and item selection. Med Care.

[B37] Kornstein SG, Schatzberg AF, Thase ME, Yonkers KA, McCullough JP, Keitner GI, Gelenberg AJ, Davis SM, Harrison WM, Keller MB (2000). Gender differences in chronic major and double depression. J Affect Disord.

[B38] Siris SG, Addington D, Azorin JM, Falloon IR, Gerlach J, Hirsch SR (2001). Depression in schizophrenia: recognition and management in the USA. Schizophr Res.

[B39] Birchwood M, Mason R, MacMillan F, Healy J (1993). Depression, demoralization and control over psychotic illness: a comparison of depressed and non-depressed patients with a chronic psychosis. Pschol Med.

[B40] Tollefson GD, Andersen SW, Tran PV (1999). The course of depressive symptoms in predicting relapse in schizophrenia: a double blind, randomized comparison of olanzapine and risperidone. Biol Psychiatry.

[B41] Keck PE, Starkowski SM, McElroy SL (2000). The efficacy of atypical antipsychotics in the treatment of depressive symptoms, hostility, and suicidality in patients with schizophrenia. J Clin Psychiatry.

[B42] Allison DB, Mackell JA, McDonnell DD (2003). The impact of weight gain on quality of life among persons with schizophrenia. Psychiatr Serv.

[B43] Fetter JC (2003). Weight gain and quality of life among patients taking antipsychotics. Psychiatr Serv.

[B44] Wirshing DA, Wirshing WC, Kysar L, Berisford MA, Goldstein D, Pashdag J, et Mintz J, Marder SR (1999). Novel antipsychotics: comparison of weight gains liabilities. J Clin Psychiatry.

[B45] Fakhoury WK, Wright D, Wallace M (2001). Prevalence and extent of distress of adverse effects of antipsychotics among callers to a United Kingdom National Mental Health Helpline. Int Clin Psychopharmacol.

[B46] Krypsin-Exner W (1947). Beitrage zum verlauf des kopergewichtes bei psychosen. Wiener Klin Wschr.

[B47] Ascher-Svanum H., Stensland MD, Kinon BJ, Tollefson GD (2004). Weight Gain and Improvement in Psychopathology During Treatment of Schizophrenia with Antipsychotics and with Placebo [abstract]. Biol Psychiatry.

[B48] Strassing M, Brar JS, Ganguli R (2003). Body mass index and quality of life in community-dwelling patients with schizophrenia. Schizoph Res.

[B49] Kinon BJ, Basson BR, Gilmore JA, Tollefson GD (2001). Long-term olanzapine treatment: weight change and weight-related health factors in schizophrenia. J Clin Psychiatry.

[B50] Ganguly R, Kotzan JA, Miller LS, Kennedy K, Martin BC (2004). Prevalence, Trends, and Factors Associated With Antipsychotic Polypharmacy Among Medicaid-Eligible Schizophrenia Patients, 1998–2000. J Clin Psychiatry.

[B51] Leslie DL, Rosenheck RA (2002). From conventional to atypical antipsychotics and back: dynamic processes in the diffusion of new medications. Am J Psychiatry.

[B52] Awad AG, Voruganti LN (2004). Body weight, image and self-esteem evaluation questionnaire: development and validation of a new scale. Schizophr Res.

